# Liver X receptor α is essential for the capillarization of liver sinusoidal endothelial cells in liver injury

**DOI:** 10.1038/srep21309

**Published:** 2016-02-18

**Authors:** Yan Xing, Tingting Zhao, Xiaoyan Gao, Yuzhang Wu

**Affiliations:** 1 Institute of Immunology, PLA, Third Military Medical University, Chongqing, 400038, PR China; 2 Department of anatomy, Third Military Medical University, Chongqing, 400038, China

## Abstract

Liver X receptors (LXRs) play essential roles in lipogenesis, anti-inflammatory action and hepatic stellate cells (HSCs) activation in the liver. However, the effects of LXRs on the capillarization of liver sinusoidal endothelial cells (LSECs) in liver fibrosis remain undetermined. Here, we demonstrated that LXRα plays an important role in LSECs capillarization in a manner that involved Hedgehog (Hh) signaling. We found that LXRα expression in LSECs was increased in the carbon tetrachloride (CCl_4_)-induced fibrosis model. LXRα deletion markedly exacerbated CCl_4_-induced lesions assessed by histopathology, as well as inflammation and collagen deposition. Furthermore, capillarization of the sinusoids was aggravated in CCl_4_ -treated LXRα-deficient mice, as evidenced by increased CD34 expression, the formation of continuous basement membranes and aggravation of the loss of fenestrae. *In vitro*, LXR agonist could maintain freshly isolated LSECs differentiation on day 3. Furthermore, LXRα deletion led to increased expression of Hedgehog (Hh)-regulated gene in LSECs in the injured liver. Conversely, the LXR agonist could inhibit the Hh pathway in cultured LSECs. These responses indicated that LXRα suppressed the process of LSECs capillarization by repressing Hh signaling. Overall, our findings suggest that LXRα, by restoring the differentiation of LSECs, may be critical for the regression of liver fibrosis.

Liver sinusoidal endothelial cells (LSECs) account for approximately half of the non-parenchymal cells in the liver^1^. These cells are flattened and have fenestrations. LSECs are important components of the liver innate immune system, which also includes Kupffer cells and dendritic cells^2^. LSECs lose their fenestrations and develop an organized basement membrane during a process known as capillarization or defenestration that is triggered by the formation and deposition of extracellular matrix in the space of Disse, which results in LSECs dysfunction^3^. Defenestration occurs prior to the development of fibrosis and has been observed in several diseases, such as hepatitis, primary biliary cirrhosis, viral infections and non-alcoholic fatty liver disease^4^,^5^,^6^,^7^,^8^. Evidence exists that the LSECs undergo the loss of the highly specialized differentiated phenotype before activation of the HSCs and the development of fibrosis. Restoration of differentiation to LSECs was shown to lesd to HSCs quiescence and the regression of fibrosis^9^,^10^.

The liver X receptors (LXRs), LXRα and LXRβ, are nuclear receptors that belong to the large family of ligand-activated transcription factors. The natural ligands for LXRs are oxysterols, and two non-steroidal synthetic compounds, GW3965 and T0901317 (T0), have also been identified as LXRs agonists. In addition to their importance in metabolic pathways including cholesterol, glucose, bile acid, lipid and carbohydrate metabolism, LXRs have emerged as important regulators of both innate immunity and acquired immunity^11^. Previous studies have shown that LXRs influence the activation of HSCs and contribute to the pathogenesis of liver fibrosis^12^, but a role for LXRs in the capillarization of LSECs, which precedes hepatic fibrosis, has not yet been elucidated. Analysis of the expression patterns of these receptor subtypes has shown that LXRα is mainly expressed in tissues with high lipid metabolism such as liver, intestine, adipose tissue and macrophages, LXRβ is expressed ubiquitously^13^,^14^. Furthermore, previous studies have suggested that LXRα is associated with hepatic inflammation and fibrosis in human non-alcoholic fatty liver disease (NAFLD)^15^,^16^.

This study aimed to evaluate the correlation between LXRα and the capillarization of LSECs associated with fibrosis. We found that LSECs in LXRα−/− mice were more susceptible to capillarization induced by CCl_4_. Subsequently, T0 was evaluated for its protection against the development of spontaneous LSECs capillarization *in vitro*. As Hedgehog (Hh) signaling has been shown to play a crucial role in regulating LSECs capillarization and to be regulated by the LXRs^17^, the involvement of Hh signaling underlying the protective effects by LXRα was also explored.

## Results

### LXRα expression in LSECs increases in response to CCl_4_ administration

**Figure 1:**
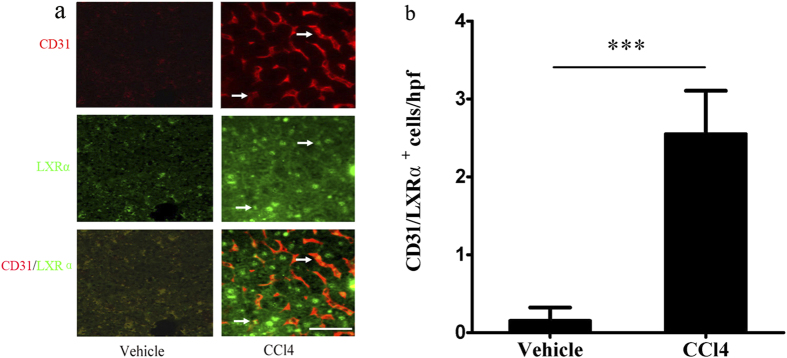
LXRα expression in LSECs of WT mice increased in response to CCl_4_ administration. (**a**) Double immunofluorescence for the CD31 (red) and LXRα (green), White arrows in the upper panel indicated activated LSECs; white arrows in the middle panel indicated LXRα-positive cells; white arrows in the bottom indicated the CD31/ LXRα double positive cells. Scale bar, 50 μm. (**b**) Statistical analysis of the number of CD31/ LXRα double positive cells in the liver from CCl_4_ treated mice or vehicle -treated controls. Data are presented as mean ± SD (***p < 0.001, n = 4, Student’s t-test).

Double immunostaing for CD31 (a marker of capillarised LSECs^10^) and LXRα was used to detect the change of LXRα expression in LSECs from WT mice after CCl_4_ administration. The number of CD31 and LXRα double-positive sinusoidal cells was negligible in the vehicle-treated mice, but double-positive sinusoidal cells were increased in the CCl_4_-treated group (*p* < 0.001; Fig. 1a,b). These results indicate that LXRα may be related to the capillarization of LSECs and hepatic fibrosis.

### Aggravation of liver injury and fibrogenesis in LXRα KO (knockout) mice after CCl_4_ treatment

**Figure 2:**
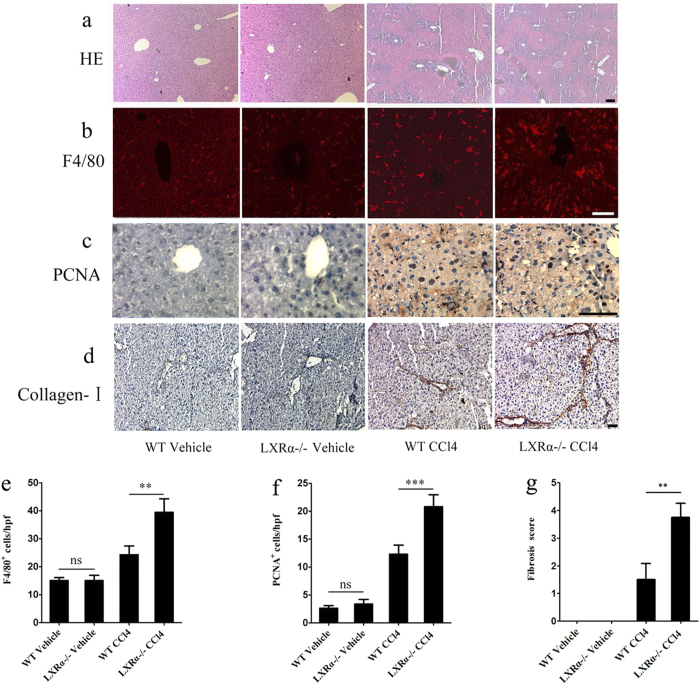
Aggravated liver injury in LXRα KO mice treated with chronic CCl_4_ treatment. (**a**) HE staining of livers with chronic CCl_4_ treatment. Scale bar, 50 μm. (**b**) F4/80 immunostaining of livers with chronic CCl_4_ treatment. Scale bar, 50 μm. (**c**) PCNA immunohistochemical staining of livers with chronic CCl_4_ treatment. Scale bar, 50 μm. (**d**) Collagen α1 (**I**) immunohistochemical staining of livers with chronic CCl_4_ treatment. Scale bar, 50 μm. (**e**) Statistical analysis of the number of F4/80 positive cells. Data are presented as mean ± SD (**p < 0.01, n = 4, Student’s t-test). (**f**) Statistical analysis of the number of PCNA positive cells. Data are presented as mean ± SD (***p < 0.001, n = 4, Student’s t-test). (**g**) Liver fibrosis score was quantified. Data are presented as mean ± SD (**p < 0.01, n = 4, Student’s t-test).

We investigated hepatic histopathology after chronic CCl_4_ treatment by hematoxylin and eosin (HE) staining. By histology, neither wild-type (WT) nor LXRα−/− livers were affected by the injection of vehicle and they presented a normal architecture. In the CCl_4_-treated WT mice, the livers showed hepatocytes necrosis and extensive disorganization with the beginning of septa formation. CCl_4_-treated LXRα−/− livers showed more septa formation, which was characterized by more severe hepatocytes necrosis, inflammatory cells infiltration and congestion (Fig. 2a).

F4/80 staining was used to observe the infiltration of macrophages and activation of Kupffer cells in the liver after chronic injury. The population of F4/80^+^ cells was greatly increased in the CCl_4_-treated LXRα−/− mice compared with that in the CCl_4_-treated WT mice (*p* < 0.01; Fig. 2b,e). Furthermore, there was no significant difference in the number of F4/80^+^ cells in the liver between vehicle-treated WT and vehicle-treated LXRα−/− mice (*p* > 0.05; Fig. 2b,e).

We next evaluated the effect of LXRα on hepatocyte proliferation following chronic CCl_4_ administration. Proliferating cell nuclear antigen (PCNA)-positive hepatocytes in WT mice were significantly increased after CCl_4_ injection. Compared with the CCl_4_-treated WT mice, PCNA-positive hepatocytes were dramatically increased in the CCl_4_-treated LXRα−/− mice (*p* < 0.001; Fig. 2c,f). Very few PCNA-positive hepatocytes could be observed in both vehicle-treated WT and vehicle-treated LXRα−/− mice (*p* > 0.05; Fig. 2c,f). These results suggest that the proliferative capability of hepatocytes after CCl_4_ administration is enhanced by the genetic deletion of LXRα.

To examine the influence of LXRα expression on liver fibrosis, chronic CCl_4_ treatment was used. Collagen α1 (I) immunohistochemical staining showed that the deposition of collagen-I was increased in the portal tracts, septa and perisinusoidal spaces in WT mice treated with CCl_4_; whereas LXRα−/− mice treated with CCl_4_ showed aggravated collagen accumulation in the liver (*p* < 0.01; Fig. 2d,g). Together, these data show that LXRα deletion aggravates inflammation and fibrosis in the liver injury.

### Exacerbation of hepatic sinusoidal capillarization in LXRα KO mice after chronic CCl_4_ administration

**Figure 3:**
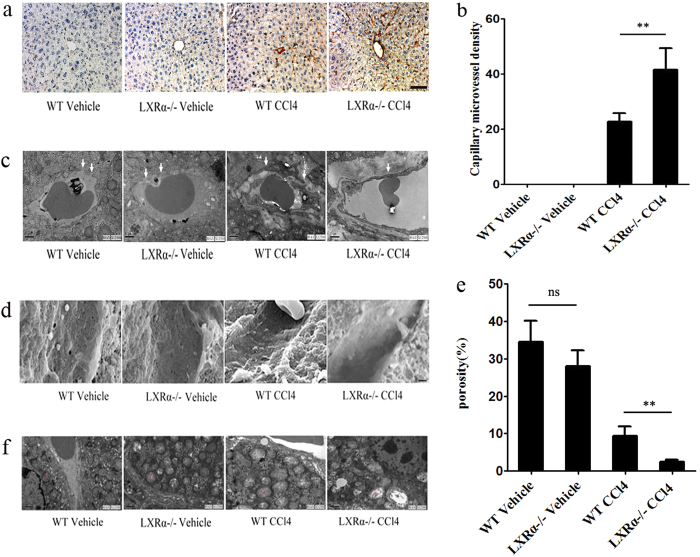
Exacerbation of LSECs capillarization in LXRα KO mice induced by CCl_4_ chronic administration. (**a**) Immunohistochemical staining for CD34 in livers. Scale bar, 50 μm. (**b**) Statistical analysis of capillary MVD in livers. Data are presented as mean ± SD (**p < 0.01, n = 4, Student’s t-test). (**c**) TEM and (**d**) SEM of hepatic sinusoids from WT and LXRα KO mouse treated with CCl_4_. White arrows indicate the change of basement membranes in hepatic sinusoids. Scale bar, 1μm. (**e**) Statistical analysis of porosity. Data are presented as mean ± SD (**p < 0.01, n = 3, Student’s t-test). (**f**) Ultrastructure of mitochondria (by TEM) in hepatocytes from WT and LXRα KO mouse treated with CCl_4_. Abbreviations: m, mitochondrion.

CD34 is not expressed by endothelial cells of normal liver sinusoids, but is expressed after liver sinusoidal capillarization has developed. CD34 expression in areas of sinusoidal capillarization (capillary microvessel density [MVD]) was examined according to Weidner *et al.*^18^. In the CCl_4_-treated WT mice, there were areas where sinusoidal endothelial cells were diffusely positive for CD34 (Fig. 3a). More CD34-positive cells were found in the CCl_4_-treated LXRα−/− mice (*p* < 0.01; Fig. 3a,b). No CD34 expression was detected in either vehicle-treated WT or LXRα−/− mice (Fig. 3a,b).

Transmission electron microscopy (TEM) of the sinusoidal areas of hepatic tissues showed that in both groups of vehicle-treated mice, the sinusoids contained numerous fenestrae with a discontinuous basement membrane that could be observed on the basal side of LSECs. In the LXRα−/− mice treated with CCl_4_, the deposition of extracellular matrix proteins in Disse’s spaces was found, with continuous basement membranes evident on the basal side of LSECs. However, CCl_4_-induced changes in sinusoidal areas could be ameliorated in WT mice (Fig. 3c).

Scanning electron microscopy (SEM) was used to evaluate the sinusoidal lining. As shown in Fig. 3d, in sinusoidal areas of livers from WT mice treated with CCl_4_, fenestrae of LSECs were markedly decreased in number. In the CCl_4_-treated LXRα−/− mice, LSECs fenestrae had completely disappeared, whereas in both groups of vehicle-treated mice, a thin sinusoidal lining containing many fenestrae could be observed (*p* < 0.01; Fig. 3d,e). Collectively, these data indicate that LXRα deletion increases susceptibility to liver injury, which is at least in part, triggered by LSECs capillarization.

In addition, TEM results revealed changes in mitochondrial structure and the shape of hepatocytes. LXRα−/− mice displayed mitochondrial swelling and loss of cristae compared with mitochondria from the WT mice. CCl_4_ induced more serious damage in mitochondria from WT mice. Thus, hepatocytic mitochondria injuries were aggravated in the CCl_4_-treated LXRα−/− mice, including aggravated mitochondrial swelling, loss of cristae and vacuole-like changes (Fig. 3f). These findings reveal that LXRα play an important role in mitochondrial injuries, which is closely associated with the liver injuries.

### LXRα inhibits LSECs capillarization via Hh signaling *in vivo*

**Figure 4:**
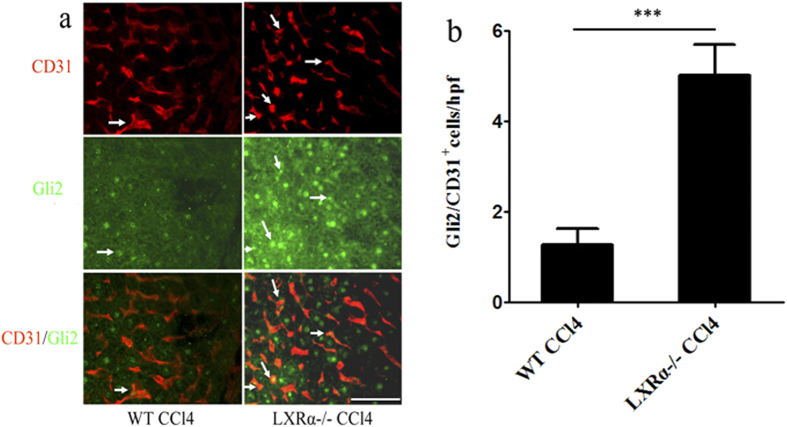
LXRα inhibits LSECs capillarization via Hh signalling *in vivo*. (**a**) Double- stained for the Hh-target gene, Gli2 (green), and the LSECs activation marker, CD31 (red). White arrows indicate the CD31/Gli2 double positive cells. Scale bar, 50 μm. (**b**) Statistical analysis of the number of Gli2/CD31 double-positive cells. Data are presented as mean ± SD (***p < 0.001, n = 4, Student’s t-test).

Hh ligands are scarce in healthy adult livers, but liver injury increases the production of Hh ligands. Thus, Hh signaling becomes dramatically activated in injured livers. It has been reported that Hedgehog signaling might be an important factor that is involved in liver injury, liver fibrosis and LSECs capillarization^17^. Negative regulation of Hh signaling by LXR has been demonstrated^19^. The role of LXR in the regulation of liver fibrosis-associated capillarization of the sinusoids may exert via Hedgehog signaling. To examine this hypothesis, Hh pathway activity was investigated in liver injury induced by CCl_4_. Double immunofluorescence for Gli2 and CD31 (a marker of capillarised LSECs) was used to assess the expression of the Hh target gene Gli2 in LSECs. LSECs in WT mice treated with CCl_4_ consistently expressed Gli2, but treatment of LXRα−/− mice with CCl_4_ significantly increased the number of Gli2 -positive cells in LSECs (*p* < 0.001; Fig. 4a,b), indicating that LXRα may modulate the Hh pathway in the process of LSECs capillarization.

### LXR agonist inhibits LSECs capillarization via Hh signaling *in vitro*

**Figure 5:**
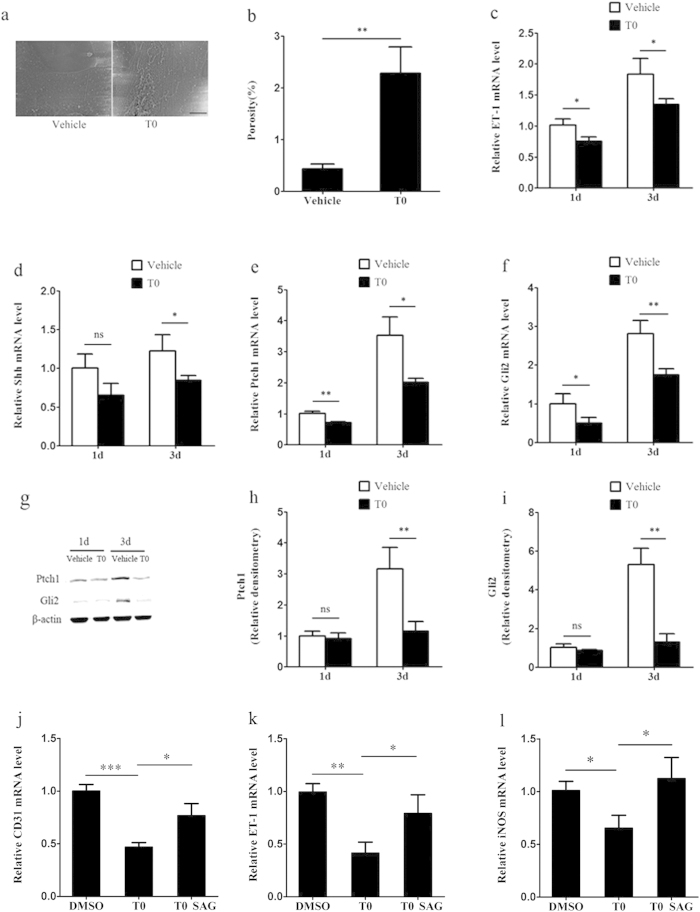
T0 inhibits LSECs capillarization via Hh signalling *in vitro*. (**a**) SEM of LSECs cultured with and without T0 for 2 days showed loss of fenestrae in sieve plates *in vitro* in the absence of T0 and maintenance of fenestrae with T0. Scale bar, 5μm. (**b**) Porosity of liver sections was quantified. Data are presented as mean ± SD (**p < 0.01, n = 3, Student’s t-test). (**c**) Quantitative Real-Time PCR analysis of ET-1 expression changes during culture-induced capillarisation with and without T0. Data are presented as mean±SD (*p < 0.05, n = 3). (**d**–**f**) Quantitative Real-Time PCR analysis of the expression change of Shh ligand, the cell surface receptor for Hh ligand Ptch1 and Hh target gene Gli2. Data are presented as mean ± SD (*p < 0.05, **p < 0.01, n = 3, Student’s t-test). (**g–i**) Western blot and graphic analysis of the expression of Ptch1 and Gli2 in the cultured LSECs with and without T0. The levels of the Ptch1 and BLBP protein are expressed relative to the levels of the β-actin. Data are presented as mean ± SD (**p < 0.01, n = 3, Student’s t-test). Abbreviations:d,day. (**j–l**) LSECs were treated with T0 for 24 hours and then treated with either DMSO or SAG for 24 hours. Quantitative Real-Time PCR analysis of the gene expression changes of iNOS, ET-1 and CD31. Data are presented as mean ± SD (*p < 0.05, **p < 0.01, ***p < 0.001, n = 3, Student’s t-test).

To more directly test the hypothesis that we have proposed above, we isolated LSECs from WT mice and cultured with or without T0. We examined the beneficial effects that LXR agonist may have exerted on LSECS *in vitro*. Fenestration of LSECs was rapidly lost after 2 days of culture, but fenestration was maintained in the presence of 5 μM T0 on day 3 (*p* < 0.01; Fig. 5a,b). Accordingly, mRNA expression of ET-1, a capillarization- associated gene, was demonstrated in cultured LSECs. ET-1 mRNA levels decreased by approximately 25% and 27% after treatment with T0 compared with vehicle on day 1 and day 3 of culture, respectively (*p* < 0.05; Fig. 5c).

LSECs capillarization was inhibited by T0 after 2 days of culture, and this process was accompanied by inactivation of the Hh pathway. The mRNA expression levels of Shh ligand, the cell surface receptor for Hh ligand Patched1 (Ptch1) and the Hh target gene Gli2 were assessed in LSECs on day 1 or 3 of culture.T0 treatment inhibited the mRNA expression of Ptch1 and Gli2 on day 1 (Shh: *p* > 0.05; Ptch1: *p* < 0.01; Gli2: *p* < 0.05) and inhibited Shh, Ptch1 and Gli2 mRNA level on day 3 (Shh: *p* < 0.05; Ptch1: *p* < 0.05; Gli2: *p* < 0.01), indicating that LXR regulated the Hh pathway activity while capillarization progressed (Fig. 5d-f). The protein levels of Ptch1 and Gli2 were further confirmed by western blot. Decreased levels of Ptch1 and Gli2 on day 3 were induced by T0 treatment (Ptch1: *p* < 0.01; Gli2: *p* < 0.01), but there was no obvious difference on day 1 (Ptch1: *p* > 0.05; Gli2: *p* > 0.05; Fig. 5g–i).

To further confirm that the effects of T0 on LSECs capillarization were mediated by Hh signaling, primary LSECs were treated with T0 along with 300 nM Hh agonist SAG (a potent agonist of Hh signaling that activates the Smoothened (Smo) protein function)^20^. The effect of T0-induced down-regulation expression of LSECs capillarization markers (inducible nitric oxide synthase (iNOS), ET-1 and CD31) was blocked by SAG (Fig. j–l).

Therefore, these results show that the LXR plays an important role in the maintenance of LSECs fenestration by regulating Hh pathway activity.

### LXR agonist attenuates CCl_4_-induced liver injury, but induces the exacerbation of hepatic steatosis *in vivo*

**Figure 6:**
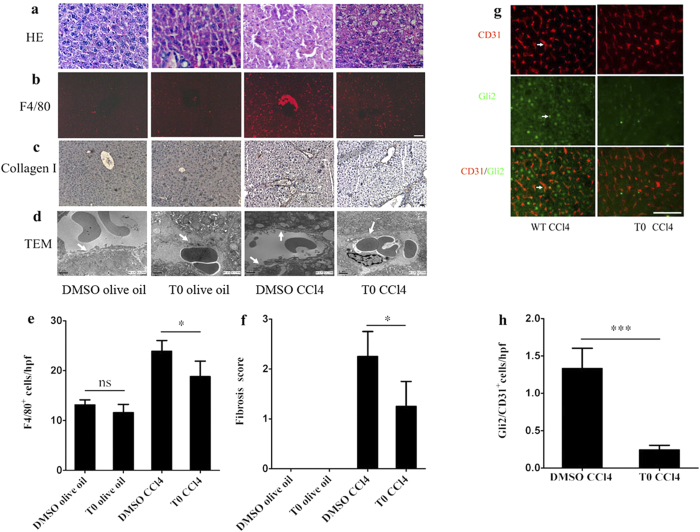
T0 attenuated CCl_4_-induced liver injury, but induced exacerbation of hepatic steatosis *in vivo*. (**a**) HE staining of livers in the groups: DMSO and olive oil; T0 and olive oil; DMSO and CCl_4_; T0 and CCl_4_. Scale bar, 50 μm. (**b**) F4/80 immunostaining of livers in the groups: DMSO and olive oil; T0 and olive oil; DMSO and CCl_4_; T0 and CCl_4_. Scale bar, 50 μm. (**c**) Collagen α1(I) immunohistochemical staining of livers in the groups: DMSO and olive oil; T0 and olive oil; DMSO and CCl_4_; T0 and CCl_4_. Scale bar, 50 μm. (**d**) TEM of hepatic sinusoids from the groups: DMSO and olive oil; T0 and olive oil; DMSO and CCl_4_; T0 and CCl_4_. White arrows indicate the change of basement membranes in hepatic sinusoids. Scale bar, 1μm. (**e**) Statistical analysis of the number of F4/80 positive cells. Data are presented as mean±SD (*p < 0.05, n = 4, Student’s t-test). (**f**) Liver fibrosis score was quantified. Data are presented as mean ± SD (*p < 0.05, n = 4, Student’s t-test). (**g**) Double immunstaing for the expression of the Hh-target gene, Gli2 (green), and the LSECs activation marker, CD31 (red). White arrows indicate the CD31/Gli2 double positive cells. Scale bar, 50 μm. (**h**) Statistical analysis of the number of Gli2/CD31 double-positive cells. Data are presented as mean±SD (***p < 0.001, n = 4, Student’s).

To evaluate the possible protective effects afforded by a LXR agonist in CCl_4_-induced liver injury, T0 pretreatment *in vivo* was employed. HE staining showed that the control DMSO and olive oil- treated mice had livers with a normal architecture, while the T0-treated group showed some lipid droplets in the liver. The livers of mice treated with CCl_4_ showed necrotic areas that were characterized by severe hepatocytes necrosis and inflammatory cells infiltration. Although these findings could also be observed in the T0 and CCl_4_-treated group, the incidence and severity of histopathological lesions were reduced compared with those in the CCl_4_-treated group. However, lipid droplets were also observed in T0 and CCl_4_-treated group (Fig. 6a).

In the livers of mice treated with CCl_4_, the population of F4/80^+^ cells was greatly increased compared with that in the control DMSO and olive oil- treated mice, and T0-pretreatment decreased the number of F4/80^+^ cells after CCl_4_ injection (*p* < 0.05; Fig. 6b,e). Furthermore, there was no significant difference in the number of F4/80^+^ cells in the liver between the control and T0 -treated groups (p > 0.05; Fig. 6b,e).

Collagen-I was not expressed in the control or T0- treated groups. Deposition of collagen-I could be observed in the livers of mice treated with CCl_4_. By contrast, the expression of collagen-I was significantly decreased in the T0 and CCl_4_-treated group (*p* < 0.05; Fig. 6c,f).

TEM of the sinusoidal areas of livers showed that in the control and T0-treated groups, sinusoids contained numerous fenestrae, with a discontinuous basement membrane, could be observed on the basal side of LSECs. In the CCl_4_-induced sinusoidal areas, deposition of extracellular matrix proteins in the Disse’s spaces was found, with continuous basement membranes evident on the basal side of LSECs. However, the CCl_4_-induced changes in the sinusoidal areas could be ameliorated by T0 pretreatment (Fig. 6d).

Double immunostaing for Gli2 and CD31 (a marker of capillarised LSECs) was performed to assess the effects of T0 on the expression of the Hh target gene Gli2 in LSECs. In the mice treated with CCl_4_, consistent expression of Gli2 in capillarised LSECs was observed. T0 pretreatment could reduce the number of Gli2 and CD31 double-positive cells in the liver (*p* < 0.001; Fig. 6g,h).

Theses results indicate that activation of LXRs attenuate CCl_4_-induced liver inflammation, fibrosis and hepatic sinusoidal capillarization by inhibiting Hh signaling. However, LXR activation can induce hepatic lipogenesis.

## Discussion

LXRα plays a crucial role in hepatic lipogenesis and steatosis. The mRNA and protein levels of LXRα as well as its downstream lipogenic genes are aberrantly increased in NAFLD, hepatic fibrosis and HCV patients^15^,^16^. Our results also confirmed that LXRα is related to hepatic fibrosis in an experimental animal model. LXRs activation can inhibit HSCs activation; whereas LXRαβ deletion enhances the activation of HSCs and exacerbates CCl_4_-induced liver fibrosis^12^. In addition, LXRs inhibit skin fibrosis by suppressing of macrophage infiltration and decreasing release of the pro-fibrotic cytokine IL-6^21^. LXRs have been identified as a novel target for antifibrotic therapy. Although our findings support the antifibrotic role of LXRs, we may have established an indirect antifibrotic mechanism of LXRα that involves LSECs capillarization associated-fibrosis. In this study, we demonstrated that LXRα deletion aggregated liver injury, fibrogenesis and LSECs capillarization *in vivo*, while T0 treatment resulted in the inhibition of LSECs capillarization *in vitro*. We also found that LXRα regulated Hh signaling in the process of LSECs capillarization.

LSECs provide a porous barrier that facilitates access of hepatocytes to oxygen and small molecules in the microcirculation. LSECs clear colloids and macromolecules, promote HSCs quiescence, and induce immune tolerance. Both *in vitro* and *in vivo* studies have shown that LSECs maintain HSCs quiescence and induce the reversion of activated HSCs to quiescence^9^,^10^. When LSECs dedifferentiate to a defenestrated “capillarized” phenotype, HSCs become activated. LSECs capillarization precedes fibrosis in both human chronic liver diseases and experimental animal models. Moreover, reversal of capillarization promotes reversion of HSCs quiescence and amelioration of fibrosis^9^. We demonstrated that LXRα deletion aggravated fibrosis-associated sinusoidal capillarization in mice treated with CCl_4_, providing further evidence of a link between LSECs and the progression of liver fibrosis *in vivo*.

In this present study, hepatocyte proliferation was enhanced in LXRα knock out mice compared with WT mice after CCl_4_-induced liver injury, suggesting that LXRα is involved in hepatocyte proliferation. This was determined by the increased immunohistochemical staining of PCNA in hepatocytes. Consistent with previous studies, Zhang X *et al.* found that cytosolic sulfotransferase 2B1b (SULT2B1b) and its product, 5-cholesten-3β-25-diol-3-sulfate (25HC3S) as LXRs antagonist, induced hepatocytes proliferation by inactivating oxysterol/LXR signaling^22^,^23^. Furthermore, activation of LXR in the regenerating liver in response to partial hepatectomy reduced hepatocyte proliferative capability^24^. Collectively, our data suggest that LXRα plays a physiological role in hepatocyte proliferation.

One effect of LXRα is to cause changes in mitochondrial morphology and the structure of hepatocytes. TEM examination revealed that LXRα deletion induced mitochondrial swelling and the disruption of normal cristae morphology. Moreover, in LXRα−/− mice, treatment with CCl_4_ aggravated mitochondrial injury. Consistent with this study, Qing *et al.* found that LXRα knockout mice exhibited exacerbated mitochondrial dysfunction^25^ and T0 attenuated high glucose-induced mitochondrial damage and apoptosis in cardiomyocytes^26^. Based on these studies, we may infer that the role of LXRα in mitochondrial morphology, structure and function is related to its effects on inflammation and metabolism, although this prediction need to be further confirmed in the future studies.

Hh signaling is crucial for repair of the adult liver^27^. In healthy adult livers, Hh ligands are scarce, but increase local production in liver injury. Hh signaling becomes greatly activated in injured livers, because several cell types, such as HSCs and various immune cells, produce and respond to Hh ligands^28^,^29^. *In vitro* study has confirmed that LSECs expressed and responded to Hh ligands and that capillarization development was regulated by Hh signaling^17^. Another study has confirmed the interaction between LXRs and Hh pathway in the differentiation of bone marrow stromal cells (MSCs). Activation of LXRs has been shown to suppress the expression of Hh-target genes, Ptch1, Gli1, and Gli-dependent transcriptional activity and regulated (MSCs) differentiation into osteoblasts^19^. Our present findings demonstrated that LXRα deletion increased the expression of Hh-target gene *in vivo*, while T0 inhibited upregulation of mRNA and protein levels of Hh signaling components *in vitro*. Furthermore, Hh agonist SAG blocked the inhibiting effect of LSECs capillarization induced by T0. Our results indicate that the molecular mechanisms underlying the LXR-Hh pathway show “cross talk” in LSECs capillarization.

LSECs capillarization not only precedes fibrosis, but also may be an integral part of the process of fibrosis. Restoring LSECs phenotype may be a beneficial approach to regress the development of fibrosis. Our findings indicate that LXRα regulates LSECs capillarization and reverses CCl_4_-induced fibrosis simultaneously and activation of LXRα maintains LSECs differentiated phenotype. We identified a new role for LXRα in LSECs capillarization. Considering the major side effect of LXR agonists, the translation our findings into clinical practice still has a long way to go.

## Methods

### Animals

Male LXRα−/− mice on a C57/BL6 background were provided by Jan-Ake Gustafsson. All animals were housed in the temperature-controlled room with a 12 hour light/dark cycle, pathogen-free conditions, and free access to water and standard chow. To induce chronic liver injury by carbon tetrachloride (CCl_4_) as described previously^12^, intraperitoneal injection of a 10% solution of CCl_4_ in sterile olive oil (0.5 μl pure CCl_4_/g body weight) was used biweekly for a month, with harvesting 72 hours after the last dose. Control groups received the same volume of vehicle (olive oil) intraperitoneally.

The evaluation *in vivo* of a possible protective effect was afforded by LXR agonist T0901317 (Cayman Chemical Co., Ann Arbor, MI, USA). Male C57/BL6 mice were randomly divided into the following four groups: (1) dimethyl sulfoxide (DMSO) and olive oil group, (2) T0 and olive oil group, (3) DMSO and CCl_4_ group and (4) T0 and CCl_4_ group. Pretreatment of T0 (dissolved in DMSO at 50 mg/ml) was intraperitoneally (i.p.) injected (50 mg/kg) daily for 7 days. Intraperitoneal injection of a 10% solution of CCl_4_ in sterile olive oil (0.5 μl pure CCl_4_/g body weight) was used biweekly for a month. The control groups received an equivalent dose of vehicle DMSO and/or olive oil. Mice were sacrificed 72 h after the last CCl_4_ or vehicle injection.

### Ethical considerations

Animal maintenance and experimental procedures were performed in accordance with the National Institutes of Health Guidelines for the Use of Experimental Animals and approved by the Ethics Committee and the Medicine Animal Care Committee of the Third Military Medical University.

### LSECs isolation and culture

LSECs were isolated from WT mice by collagenase digestion, immunomagnetic bead method as previously described with modifications^30^. The mice were anesthetized by 10% chloral hydrate and the portal vein was exposed, cannulated with a 22-gauge intravenous catheter (BD Biosciences, Franklin Lakes, NJ, USA). The liver was pre-perfused via the portal vein with liver perfusion medium (Gibco-Invitrogen, Carlsbad, CA) for 10 min and then injected with 5 mg/ml collagenase IV (Sigma-Aldrich, St. Louis, MO) in buffer (pH 7.4) containing 135 mM NaCl, 5 mM KCl, 4.8 mM Glucose, 17.6 mM HEPES, 22 mM NaHCO_3_, 2 mM CaCl_2_, 2 mM MgCl_2_ ((all chemicals obtained from Sigma-Aldrich, St. Louis, MO) for 10 min. After the two-step collagenase perfusion, the liver was disrupted and incubated in 5 mg/ml collagenase IV digestion for 15 minutes at 37 °C. The undigested tissue was removed by 100-μm nylon mesh filter (Falcon; BD Biosciences). The filtered suspension was centrifuged (70 × g) for 1 min for three times to remove hepatocytes, and the supernatant was centrifuged (300 × g) for 10 min at 4 °C. The pellet was resuspended in 90 μl of buffer prepared by MACS BSA stock solution (Miltenyi Biotec, Auburn, CA, USA) and autoMACS rinsing solution (Miltenyi Biotec, Auburn, CA, USA) and incubated with 10 μl anti-CD146 (LSEC) immunomagnetic beads (Miltenyi Biotec, Auburn, CA, USA) per 10^7^cells for 15 min at 4 °C in the dark. The cells were washed and passed through a MACS column (Miltenyi Biotec, Auburn, CA, USA) according to manufacturers’ instructions. The freshly isolated cells were cultured on fibronectin (Sigma-Aldrich, St. Louis, MO)-coated culture flask in DMEM with 10% FBS, 100 IU/ ml penicillin, 100 mg/ ml streptomycin (Gibco-Invitrogen, Carlsbad, CA) and incubated in a CO_2_ incubator at 37 °C. Cells were treated with vehicle (DMSO) or 5 μM T0. To further confirm that the effects of T0 on LSECs capillarization are mediated by Hh signalling, SAG was used to activate the Hh signalling. After 24 hours of T0 treatment, cells were then treated with 300 nM SAG (Abcam, Cambridge, MA, USA) or DMSO for 24 hours. Cells were harvested for subsequent experiments. The viability of LSECs were assessed by trypan blue (Invitrogen, Carlsbad, CA, USA) exclusion. Yields of LSECs are 0.9−1.5 × 10^6^ cells/per mouse liver with a purity of approximatively 92% fenestrated cells, as determined by SEM and fluorescence activated cell sorter (FACS) for CD31 and CD146 (Supplementary Figure S1).

### Purity test by flow cytometry

The isloated LSECs were resuspended and modulated at a cell density of 5 × 10^5^/ml. Followed by incubating with Fc block (anti-CD16/32, BD Biosciences), PE-conjugated anti-CD31(clone:390) (Abcam, Cambridge, MA, USA) and FITC-conjugated anti-CD146 (clone:ME-9F1) (Miltenyi Biotec, Auburn, CA, USA) were added into 100 μl cell suspension. Then, the cell suspension was incubated for 45 min at 4 °C. After washed twice by PBS, fluorescence activated cell sorter (FACS) analysis was performed by BD FACS CanTo II and analyzed using FlowJo software (TreeStar).

### Scanning electron microscopy and quantitative imaging

Livers and LSECs were fixed in 2.5% glutaraldehyde, postfixed with 1% osmium tetroxide, dehydrated with graded alcohols, dried with hexamethyldisilazane, sputter-coated with gold and examined using a SEM (HITACHI, S-3400 N). Porosity, the percentage of LSECs surface held by fenestrae which were counted manually by an investigator blinded to the identity of the slide using Zeiss AxioVision 3.0 system. Each average was taken from 10 images and three animals were tested at each group.

### Transmission electron microscopy

Fresh specimens were fixed in 2.5% glutaraldehyde, 0.1 M phosphate buffer (pH 7.4) for 1 h and post-fixed in 1% osmium tetroxide for 4 h. After dehydration with a graded alcohols gradient, the samples were embedded in epoxy resin and cut into thin sections. Three samples per each group were examined with a TEM (FEI, TECNAI10) to observe changes in LSECs basement membranes and integrity of the space of Disse.

### Immunohistochemistry and immunofluorescence

Livers were dissected and post-fixed in 4% paraformaldehyde overnight at 4 °C. Tissues were embedded in paraffin and processed for sections (5μm). Hematoxylin and eosin staining was used to examine the histology of liver. Paraffin sections were deparaffinized in xylene, rehydrated through graded alcohol, and processed for antigen retrieval by boiling in 10 mM citrate buffer (pH 6.0) for 5 min. The sections were incubated in 0.5% H_2_O_2_ in phosphatebuffered saline for 30 min at 22 °C to quench endogenous peroxidase and then incubated in 0.5% Triton X-100 in phosphate-buffered saline for 30 min. To block non-specific binding, sections were incubated in 3% bovine serum albumin (BSA) for 1 h at 37 °C. Sections were then incubated with anti-proliferating cell nuclear antigen (PCNA) (1:500, Chemicon, Temecula, CA, USA), anti-collagen-α1(I) (1:100, Abcam, Cambridge, MA, USA) and anti-CD34 (1:100, eBioscience, San Diego, CA), antibodies in 1% BSA and 0.1% Triton X-100 overnight at 4 °C.1% BSA replaced primary antibodies in negative controls. After washing, the sections were then incubated with biotin-conjugated secondary antibodies and visualized under bright-field microscopy with a diaminobenzidene substrate kit (Vector Laboratories, Burlingame, CA, USA). For immunofluorescence, sections were incubated with anti-LXRα (1:100, kindly provided by Jan-Ake Gustafsson), anti-CD31 (1:100, Abcam, Cambridge, MA, USA), anti-F4/80 (1 : 100, eBioscience, San Diego, CA) and Gli2 (1:00, Abcam, Cambridge, MA, USA) in 1% BSA overnight at 4 °C. BSA alone served as the negative control. Secondary antibodies conjugated with Cy3 or FITC (1:200, Jackson ImmunoResearch, West Grove, PA, USA) were then added and mounted in Vectashield (Vector). The stained cells were viewed and photographed under a Zeiss (Oberkochen, Germany) Axivert microscope equipped with a Zeiss AxioCam digital color camera connected to the Zeiss AxioVision 3.0 system.

### Histological evaluation

Immunohistochemistry staining for collagen-I assessed the hepatic collagen deposition and fibrosis. The degree of fibrosis was assessed semiquantitatively by the Ishak system^31^. Fibrosis was graded as follows: stage 0—no fibrosis; stage 1—fibrous expansion of some portal areas, with or without short fibrous septa; stage 2—fibrous expansion of most portal areas, with or without short fibrous septa; stage 3—fibrous expansion of most portal areas with occasional portal to portal bridging; stage 4—fibrous expansion of portal areas with marked bridging (portal to portal as well as portal to central); stage 5—marked bridging (portal to portal and/or portal to central) with occasional nodules (incomplete cirrhosis); and stage 6—cirrhosis, probable or definite.

### Capillary microvessel density

CD34 staining for capillary microvessel density (MVD) was examined according to Weidner *et al.*^18^.Three areas of highest capillary MVD were selected in a liver section, at least five sections were counted in each mouse, and three mice were examined per group. The mean value in each group was regarded as the capillary MVD.

### Quantitative Real-Time PCR

Total RNA was extracted from LSECs using TRIzol reagent (Invitrogen, Carlsbad, CA, USA), according to the manufacturer’s instructions. Reverse transcription qPCR was performed with the Bio-Rad 5-Color System (Bio-Rad Laboratories, Hercules, CA, USA) using SYBR®Premix Ex TaqTM (Takara Bio, Dalian, China). The sequences of primers used are listed Supplementary Table S1. The conditions of qPCR were as follows: denaturation at 95 °C for 20 s followed by 40 cycles of 95 °C for 10 s,60 °C for 30 s and 72 °C for 45 s. To characterize the specificity of the qPCR products, a melting curve analysis was applied using iCycler software, and samples were run on an agarose gel. Each reaction was performed in triplicate and the mean of at least three independent experiments was calculated. The mRNA level of specific genes were normalized to β-actin and measured using the 2−ΔΔCt method^32^.

### Western blot

Cells were lysed with radioimmunoprecipitation assay (RIPA) buffer (Thermo Scientific, Rockford, IL, USA), and then the protein concentration was measured by bicinchoninic acid (BCA) protein assay with BSA as standard. Protein was submitted to electrophoresis in a 12% sodium dodecyl sulfate–polyacrylamide gel electrophoresis and electrically transferred onto a polyvinylidene difluoride transfer membrane. Membranes were blocked in 5% fat-free milk and primary antibodies were incubated overnight at 4 °C followed by peroxidase-conjugated secondary antibody incubation for 1 h at room temperature (RT). Proteins were visualized using the enhancing chemiluminescence ECL kit (Amersham Pharmacia Biotech, Piscataway, NJ, USA). The following primary antibodies were used: anti-β-actin(1 : 1000, Santa Cruz Biotechnology, Santa Cruz, CA, USA), Ptch1 (1:100, Abcam, Cambridge, MA, USA) and Gli2 (1:100, Abcam, Cambridge, MA, USA).

### Data analysis

Statistical analysis was performed using SPSS 13.0 software (SPSS Inc., Chicago, IL, USA). The data were expressed as mean ± SD. Differences between groups were determined by two-tailed unpaired Student’s t-test. A p-value less than 0.05 were considered statistically significant.

## Additional Information

**How to cite this article**: Xing, Y. *et al.* Liver X receptor α is essential for the capillarization of liver sinusoidal endothelial cells in liver injury. *Sci. Rep.* **6**, 21309; doi: 10.1038/srep21309 (2016).

## Supplementary Material

Supplementary Information
