# Supplementary material for: Liver X receptor α is essential for the capillarization of liver sinusoidal endothelial cells in liver injury
Source: Sci Rep. 2016 Feb 18;6:21309. doi: 10.1038/srep21309 (PMC4758044; doi:10.1038/srep21309)
Supplement: Supplementary Information [file srep21309-s1.doc]

Liver X receptor α is essential for the capillarization of liver sinusoidal endothelial cells in liver injury

Yan Xing^1^, Tingting Zhao^1^, Xiaoyan Gao^1^, Yuzhang Wu^1*^

1 Institute of Immunology, PLA, Third Military Medical University, Chongqing, 400038, PR China

*Corresponding author

E-mail addresses: yuzhangwu123@aliyun.com

**Supplementary Table S1.** Primer sequences for real-time PCR

β-actin 5’-GGTCCACACCCGCCACCAGTTC-3’ 5’-GGATGCCACAGGATTCCATACCC-3’

ET-1 5’- GCAGCGGTCTCTGTCTAGTG-3’

5’- CACGGCTAACTTCAGAGGCA -3’

Shh 5’-GATGTGTTCCGTTACCAGCG -3’

5’- GACTTGTCTCCGATCCCCAC-3’

Ptch1 5’-GCCGGACCGGGACTAT-3’

5’-CAACCAAAAACTTGCCGCAG-3’

Gli2 5’- GGTGTGGACTCATTGCCTGA-3’

5’-ATGCACCAAATTTACTGCCTGG-3’

iNOS 5’-CTCGGAACTGTAGCACAGCA-3’

5’-TGGAGCACAGCCACATTGAT-3’

CD31 5’-CAAGGCCAAACAGAAACCCG-3’

5’-TCGACCTTCCGGATCTCACT-3’

**Supplementary Figure S1.** Characteristics of isolated liver sinusoidal endothelial cells (LSECs) by the immunomagnetic bead method.

**
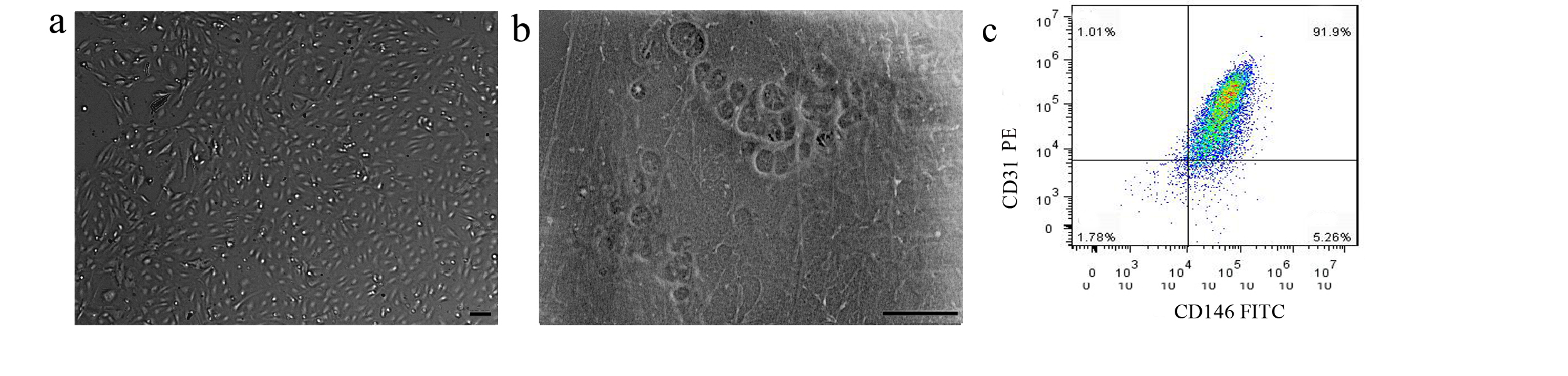
**

**Supplementary Figure S1.** Characteristics of isolated liver sinusoidal endothelial cells (LSECs) by the immunomagnetic bead method. (a) LSECs cultured for 1 day display typical ‘cobblestone’ morphology under phase contrast microscopy. Scale bar, 20μm. (b)Scanning electron microscopy (SEM) demonstrates LSECs have fenestrae grouped

into sieve plates, which is the characteristic morphology of LSECs. Scale bar, 5μm. (c) Fluorescence activated cell sorter analysis purity of freshly isolated LSECs. About 92.8% of LSECs were double-positive for CD31 and CD146.
